# *CHAT* gene polymorphism rs3810950 is associated with the risk of Alzheimer’s disease in the Czech population

**DOI:** 10.1186/s12929-018-0444-2

**Published:** 2018-05-14

**Authors:** Alice Hálová, Jana Janoutová, Laura Ewerlingová, Vladimír Janout, Ondřej Bonczek, Tomáš Zeman, Tereza Gerguri, Vladimir J. Balcar, Omar Šerý

**Affiliations:** 1 Laboratory of Neurobiology and Molecular Psychiatry, Department of Biochemistry, Faculty of Science, Masaryk University, Kotlářská 2, 611 37 Brno, Czech Republic; 2 Department of Epidemiology and Public Health, Faculty of Medicine, University of Ostrava, Ostrava, Czech Republic; 3 Laboratory of Neurobiology and Pathological Physiology, Institute of Animal Physiology and Genetics, Academy of Sciences of the Czech Republic, Veveří 97, 602 00 Brno, Czech Republic; 4 Biomolecular Modelling Laboratory, The Francis Crick Institute, London, NW1 1AT UK; 5 Discipline Anatomy and Histology and Bosch Institute, School of Medical Sciences, Sydney Medical School, The University of Sydney, Sydney, NSW 2006 Australia

**Keywords:** Alzheimer’s disease, Polymorphism, Gene, Association, Choline acetyltransferase

## Abstract

**Background:**

Cholinergic hypothesis of Alzheimer’s disease (AD) is based on the findings that a reduced and/or perturbed cholinergic activity in the central nervous system correlates with cognitive decline in patients with Alzheimer’s disease. The hypothesis resulted in the development of centrally-acting agents potentiating cholinergic neurotransmission; these drugs, however, only slowed down the cognitive decline and could not prevent it. Consequently, the perturbation of the central cholinergic signalling has been accepted as a part of the Alzheimer’s aetiology but not necessarily the primary cause of the disease. In the present study we have focused on the rs3810950 polymorphism of ChAT (choline acetyltransferase) gene that has not been studied in Czech population before.

**Methods:**

We carried out an association study to test for a relationship between the rs3810950 polymorphism and Alzheimer’s disease in a group of 1186 persons; 759 patients with Alzheimer’s disease and 427 control subjects. Furthermore, we performed molecular modelling of the terminal domain (1st-126th amino acid residue) of one of the ChAT isoforms (M) to visualise in silico whether the rs3810950 polymorphism (A120T) can change any features of the tertiary structure of the protein which would have a potential to alter its function.

**Results:**

The AA genotype of *CHAT* was associated with a 1.25 times higher risk of AD (*p* <  0.002) thus demonstrating that the rs3810950 polymorphism can have a modest but statistically significant effect on the risk of AD in the Czech population. Furthermore, the molecular modelling indicated that the polymorphism is likely to be associated with significant variations in the tertiary structure of the protein molecule which may impact its enzyme activity.

**Conclusions:**

Our findings are consistent with the results of the meta-analytical studies of the relationship between rs3810950 polymorphism and AD and provide further material evidence for a direct (primary) involvement of cholinergic mechanisms in the etiopathogenesis of AD, particularly as a factor in cognitive decline and perturbed conscious awareness commonly observed in patients with AD.

## Background

Alzheimer’s disease (AD) is a neurodegenerative disease characterised by a gradual but inexorable decline of cognitive functions such as recall and memory. AD may lead to the loss of language and social skills thus seriously disrupting the patient’s everyday life and eventually irreversibly altering his/her personality. Obviously, such condition can have a significant impact on the patient’s family and friends as well as imposing a major burden on the society as a whole. Despite major research efforts an effective cure for AD remains elusive and the treatment is, at best, symptomatic (for reviews see: [1, 2]).

Two main neuropathological findings have been correlated with AD; neurofibrillary tangles formed mainly by abnormally-phosphorylated tau proteins and senile plaques containing β-amyloid (reviews: [3, 4]). Another characteristic feature of AD is the reduction in the levels of acetylcholine (ACh) commonly found in brains of AD patients [5].

ACh was the first identified neurotransmitter (review: [6]). It is present in human brain in discreet locations mainly along the neuraxis but also in other locations which include efferent neurons targeting skeletal musculature, autonomic ganglia and sensory organs (reviews: [7, 8]). Brain cholinergic neurons are involved in the mechanisms of memory and cognition [9] and may be essential for the conscious awareness and sleep [9, 10]. This has led to the formulation of the cholinergic hypothesis of AD (review: [11]). The hypothesis may have been specifically prompted by the findings that the degeneration of cholinergic neurons could be causally linked to the cognitive decline observed in AD [12]. Subsequent clinical studies have, however, indicated that cholinergic dysfunction is characteristic of more severe forms of AD and might not always be detectable during the initial stages of the disorder even when the cognitive decline is already progressing [5]. These results would seem to suggest that the cholinergic deficit may only be a correlate, or, perhaps, a consequence, of β-amyloid toxicity and not the main cause of AD [11]. The cholinergic hypothesis of AD in its initial form thus would seem no longer tenable but it should be recognized that, at the time, the hypothesis served as a basis for the development of therapies such as those using acetylcholinesterase inhibitors [5]. These drugs remain in use to this day; they slow the cognitive decline but fail to halt the progress towards the dementia stage [13].

The role of gradually worsening cholinergic deficit in the actual progression of AD [11] is supported by findings that the choline acetyltransferase (ChAT), which is the key enzyme responsible for catalysing the synthesis of acetylcholine, is affected within the AD pathogenesis [14–17]. The patients have significantly decreased ChAT activity in the cerebral cortex and hippocampus and this seems to correlate with the severity of the dementia [18]. The role of deficient ChAT in the development of AD has been further buttressed by the findings that β-amyloid oligomers inhibit ChAT activity [19, 20].

In experiments on rats, the activity of ChAT in the hippocampus has been associated with spatial learning and memory [21]. The hippocampus is a brain region that is among the earliest and most affected by aging processes [22] and the visuospatial deficits are often the first serious manifestation of AD [23]. Furthermore, it has been claimed that overexpression of ChAT can improve cognitive functions by increasing acetylcholine levels in a rat model of Alzheimer’s disease [24].

Most of the above evidence is, however, based either on a correlation with, or, at best, a possible involvement of ChAT in the progression of AD. This does not necessarily imply causation. In contrast, if an altered risk of AD could be put to a genetic variation in a component of the cholinergic synaptic signalling i.e. be placed well upstream from the onset of the disease, the theory of a cholinergic deficit underlying causative mechanism(s) of AD would acquire more solid basis. ChAT, as the ACh-synthesising enzyme encoded by *CHAT* gene, would seem to be eminently suitable to be examined for such hypothetical association.

ChAT was discovered in rabbit brain in 1943 (for a review see [6]). The enzyme catalyses the transfer of an acetyl group from the acetyl-CoA to choline thus creating ACh. ChAT is synthesized mainly in cholinergic neurons and, for these reasons, ChAT expression in neuronal cells has been used as a marker for cholinergic system during the brain development [25, 26]. ChAT is also expressed in various types of non-neuronal cells such as muscle, immune, epithelial or endothelial cells [26]. Human *CHAT* gene is located at the chromosome position 10q11.23 and it contains several single nucleotide polymorphisms (SNP). It is the only gene encoding ChAT (EC 2.3.1.6). The first intron of the gene also carries a sequence encoding the vesicular acetylcholine transporter (VAChT, *SLC18A3*) [27].

Alternative splicing can produce several mRNA transcripts resulting in the occurrence of distinct splice variants of ChAT [28]. In humans, five splice variants, referred to as M, S, R, N1 and N2 isoforms of ChAT, have been reported. Isoforms N1 and N2 are spliced in different ways but they retain the same amino acid sequences as those in R isoform [29]. R, N1 and N2 isoforms of ChAT have molecular masses around 70 kDa and S isoform about 74 kDa. M isoform has a molecular mass about 83 kDa [29] that is caused by a 118-amino acids residue that is present only in M isoform [7, 30, 31]. M isoform is typical for primates and humans and is localized mainly in the nuclei of the cholinergic neurons whereas the 70 kDa variants are located in the cytoplasm [28, 30]. The differences in the subcellular distributions of the ChAT isoforms may reflect their roles in specific mechanisms operating in the cholinergic neurons. Moreover, there is a correlation with age and the presence and/or progression of neurodegenerative diseases such as AD. Gill et al. [30] reported that 83-kDa isoform of ChAT in young humans was localized mainly in neuronal nuclei while in older people it was also present in the perikaryal cytoplasm of the cholinergic neurons. Winick-Ng et al. [19] investigated the impact of acute exposure to oligomeric Aβ on a nuclear distribution of 83-kDa isoform of ChAT and they concluded that M isoform can influence epigenetic response in Aβ-exposed human neural cells. It would appear that the loss of the epigenetic response could contribute to the onset and progression of MCI and/or its transition to AD [10].

**Fig. 1:**
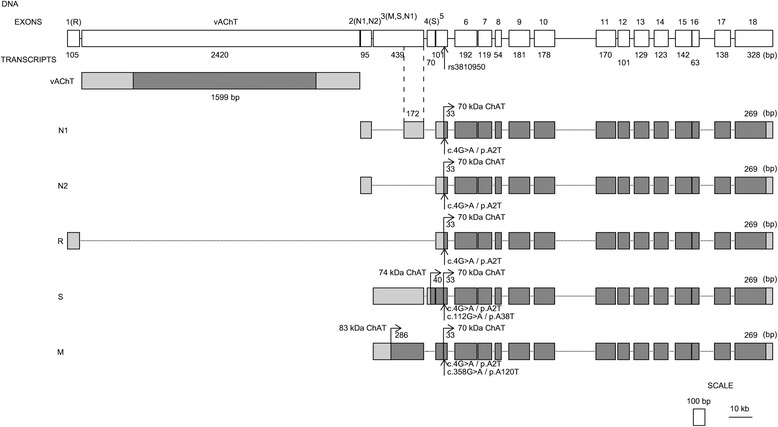
Splicing variants of CHAT gene. ChAT and VAChT (vesicular acetylcholine transporter) proteins are encoded in 18 exons of *CHAT* gene. There are 5 transcripts for ChAT (N1, N2, R, S and M) produced by alternative splicing. All transcript variants have translation initiation (start) codons for 70-kDA ChAT protein (isoform 1). The M transcript has an additional start codon for 83-kDa ChAT protein (isoform 2) and the S transcript has one for 74-kDa ChAT protein (isoform 3) (Misawa et al., 1997; Ohno et al., 2001). R, N1 and N2 transcripts are the basis for the production of same 630 amino acid-long protein product (70-kDa ChAT) where that 2nd amino acid is changed by rs3810950 polymorphism (marked by down arrow, p.A2T). In the M transcript variant of ChAT protein the 120th amino acid position is influenced by rs3810950 polymorphism (marked by down arrow, p.A120T) and in case of the S transcript it is the 38th position (marked by down arrow, p.A38T) that is influenced by rs3810950 polymorphism. White coloured boxes represent exons in the DNA sequence of the *CHAT* gene, light grey coloured boxes show untranslated regions and dark grey coloured boxes show translated regions of the mRNA (according to reference sequences NC_000010.11 and NG_011797.1, NCBI Database). The rs3810950 polymorphism and its position in mRNAs and proteins are marked by arrow up. The horizontal lines show introns and dotted lines are sequences spliced into mRNA

There are many DNA polymorphisms in the *CHAT* gene. One of the most discussed AD-related polymorphism of the *CHAT* gene has been the rs3810950 polymorphism and its putative relationship with AD [32, 33]. The missense transition of G → A in rs3810950 polymorphism results in a change of alanine to threonine [32] in the amino acid sequence. The position of the amino acid change in the isoforms M, S and R is shown in Fig. 1. The mutation is present close to the N-terminal of S (38th amino acid) and R (2nd amino acid) variants and at a distance of 120 amino acids from the N-terminal in M variant.

The objective of the present study has been to broaden and diversify the database by looking for an association between rs3810950 polymorphism of *CHAT* gene and the risk of AD in a group of Central-European patients and controls originating from the Czech Republic. The Czech population appears to be typically Caucasian [34], has been investigated in several genetic-association studies including those relevant for AD [35–37] but has never been examined for a possible link between any of the polymorphisms in ChAT gene and the risk of AD.

## Methods

### Subjects

We have examined 759 patients with Alzheimer’s disease and 427 control subjects, all originating from the Czech Republic. The two groups were, as far as possible, matched for age, gender and other characteristics (Table 1). The AD patients, hospitalized in psychiatric hospitals Jihlava, Kroměříž, Šternberk, Opava, Moravský Beroun and Ostrava, have been diagnosed according to ICD10 (International Classification of Diseases, 10th edition). Control subjects were recruited from various other departments (e.g. traumatology) in regional hospitals in Kroměříž, Olomouc and in the Faculty Hospital of Masaryk University in Brno. In the control group, the presence of dementia was ruled out using the Mini mental state examination test (MMSE; required minimum score for the control subjects was 24). Informed consent was gained from all participants or their legal representatives and formal approval for the study was granted by the Ethics Committee of the Faculty of Medicine, University of Ostrava.

**Table 1:** Physical characteristics and medical histories of AD patients and control subjects

Group	AD patients		Controls	
	Females (*n* = 589)	Males (*n* = 170)	Females (*n* = 335)	Males (*n* = 92)
Age (Year)	79.7 ± 7.5	79.3 ± 7.5	77.7 ± 7.7	78.3 ± 9.6
Body height (cm)	158.8 ± 7.3	170.7 ± 8.7	162 ± 6.6	174.6 ± 7.1
Body weight (kg)	62.3 ± 11.9	72.2 ± 12.3	72.5 ± 14.9	81.1 ± 16.6
BMI	24.5 ± 4.7	24.9 ± 3.8	27.4 ± 5.3	26.5 ± 5
Smoking	21 (3.6%)	24 (14.1%)	30 (9.0%)	19 (20.7%)
Head injury	27 (4.6%)	16 (9.4%)	25 (7.5%)	7 (7.6%)
Diabetes mellitus	165 (28.1%)	45 (26.5%)	125 (37.5%)	36 (39.1%)
Hypertension	419 (71.3%)	117 (68.8%)	260 (78.1%)	64 (69.6%)
Stroke	80 (13.6%)	24 (14.1%)	61 (18.3%)	20 (21.7%)
Cardiovascular diseases	263 (44.7%)	75 (44.1%)	143 (42.9%)	42 (45.7%)
Physical activities	216 (36.7%)	99 (58.2%)	200 (60.1%)	81 (88.0%)
CT scan	254 (43.2%)	88 (51.8%)	28 (8.4%)	7 (7.6%)

The questionnaires used for both AD patients and control subjects aimed at identifying potential lifestyle risk factors such as smoking or the lack of physical activities. Participants’ weight and height were also recorded as well as histories of other neurological, cardiovascular and cerebrovascular diseases including stroke or head injury. For obvious reasons, much of the information pertaining to the AD patients had to be sought from hospital records or by consulting patients’ next of kin and other close relatives rather than the patients themselves.

### Genetic analysis

Genomic DNA was isolated from buccal swab samples by using automatic instrument ZEPHYRUS Magneto with modified protocol as specified for BodyFluid DNA/RNA isolation kit (Elisabeth Pharmacon, Czech Republic). *CHAT* genotyping was done by polymerase chain reaction and restriction fragment length polymorphism analysis (PCR-RFLP). The fragment of interest containing the SNP was amplified using 5′-TGCAATGAGACCCCTATACAC-3′ as forward (left) primer and 5′-TCACTGCTGGGAGTTTTTGCGG-3′ as a reverse (right mismatch) primer. In the sequence of the reverse primer, the penultimate base of 3′end (adenine) was substituted by guanine to create the next cleavage site. The reaction mixture for the PCR consisted of 1 μL of DNA template (50 ng/μL), the primers were present at 0.1 nM, together with EliZyme HS FAST MIX 2× (Elisabeth Pharmacon, Czech Republic), in the final volume of 20 μL. After initial denaturation at 95 °C for 3 min, samples were amplified through 50 cycles (95 °C for 10 s, 57 °C for 30 s, 72 °C for 20 s) followed by 5 min at 72 °C; all procedure were done using Veriti thermal cycler (Applied Biosystems, USA). The resulting 234 bp PCR product was digested 30 min at 37 °C by BsuRI FD restricting enzyme (Thermo Fisher Scientific, Waltham, MA, USA) followed by deactivation of the enzyme at 80 °C for 20 min. The digested fragments were subsequently separated on a 2.5% agarose gel and stained by ethidium bromide. The obtained fragments corresponded to three amplicons of 37, 58 and 137 bp for the A allele and to four fragments of 22, 37, 58 and 117 bp for the G allele. Genotyping analysis of ApoE was performed as previously described in detail [35].

### Statistical analyses

The statistical software R (32 R Foundation for Statistical Computing, 2015 [38]) was used in all statistical analyses. Statistical significance of the putative association between the investigated polymorphism and AD was evaluated by Fisher’s exact test (Table 2). Kruskal-Wallis test was used to evaluate association between age and rs3810950 genotypes distribution. The distribution of individual genotypes versus gender was tested by Fisher’s exact test.

**Table 2:** Association between rs3810950 polymorphism and Alzheimer’s disease

Genotype/Allele	*N*		Risk	RR	Odd	OR (95% Cl)	*p*
	AD	Controls					
GG	382	229	0.63	–	1.67	–	–
GA	303	177	0.63	1	1.71	1.02 (0.8–1.31)	0.8501
AA	73	19	0.79	1.25	3.84	2.3 (1.35–3.91)	0.0015
G	1067	635	0.63	–	1.68	–	–
A	449	215	0.68	1.08	2.09	1.24 (1.03–1.5)	0.0251

*N* number of subjects, *OR* odds ratio, *CI* confidence interval

### Molecular modelling

The N-terminal domains (1st-126th residue) of the M isoform of ChAT non-mutant protein and mutant A120T of the same isoform were prepared using ab initio modelling Robetta server (http://robetta.bakerlab.org/) and homology modelling software MODELLER [39]. Search for hydrogen bond network was done via VMD [40], Schrodinger Maestro software (Schrodinger, L. Biologics Suite 2017–1) and PyMOL (The PyMOL Molecular Graphics System, Version 2.0 Schrödinger, LLC.) as well as the visualization device.

## Results

We have found statistically significant association between the risk of AD and the polymorphism rs3810950; the subjects with AA genotype had 1.25 times higher risk of AD than subjects with GA or GG genotype (*p* <  0.002, Table 2). Additionally, we have found a statistically significant association between AD and the genotype combining certain ApoE haplotypes with particular alleles in the rs3810950 polymorphism (Table 3). Among such significant associations, the ApoE haplotype E3E3 combined with the rs3810950 genotype AA and the ApoE haplotype E3E4 combined with the rs3810950 genotype GG displayed the greatest risk of AD with risk ratios (RR) 1.54 (*p* = 0.001) and 1.52 (*P* < 0.0001), resp. (Table 3).

**Table 3:** Association between ApoE polymorphism, rs3810950 polymorphism and Alzheimer’s disease

ApoE/rs3810950	*N*		Risk	RR	Odd	OR (95% Cl)	*p*
	AD	Controls					
E3/E3 GG	182	157	0.54	–	1.16	–	–
E3/E3 GA	141	99	0.59	1.09	1.42	1.22 (0.87–1.7)	0.2354
E3/E3 AA	29	6	0.83	1.54	4.83	4.16 (1.68–10.28)	0.0010
E3/E4 GG	132	29	0.82	1.52	4.55	3.92 (2.49–6.18)	< 0.0001
E3/E4 GA	118	47	0.72	1.33	2.51	2.16 (1.45–3.22)	0.0002
E3/E4 AA	23	7	0.77	1.43	3.29	2.84 (1.19–6.8)	0.0202
E4/E4 GG	16	6	0.73	1.35	2.67	2.3 (0.88–6.02)	0.1202
E4/E4 GA	10	2	0.83	1.54	5	4.31 (0.93–19.97)	0.0726
E4/E4 AA	7	1	0.88	1.63	7	6.03 (0.73–49.55)	0.0759
E2/E2 GG	3	1	0.75	1.39	3	2.59 (0.27–25.15)	0.6275
E2/E3 GG	26	29	0.47	0.87	0.9	0.78 (0.44–1.38)	0.3865
E2/E3 GA	16	23	0.41	0.76	0.7	0.6 (0.31–1.18)	0.1752
E2/E3 AA	9	5	0.64	1.19	1.8	1.55 (0.51–4.72)	0.5865
E2/E4 GG	20	6	0.77	1.43	3.33	2.87 (1.12–7.32)	0.0242
E2/E4 GA	13	4	0.76	1.41	3.25	2.8 (0.89–8.76)	0.0814
E1/E2 GG	2	1	0.67	1.24	2	1.72 (0.15–19.15)	1
E1/E4 GG	1	0	1	1.85	Inf	Inf	1

*N* number of subjects, *OR* odds ratio, *CI* confidence interval

There was no significant age bias in the frequency of genotypes, either in the AD patient group (*p* = 0.50) or in the controls (*p* = 0.59). Nor was there any statistically significant association between rs3810950 genotypes and the gender, whether in AD patients or in control subjects (*p* = 0.20 and 0.86, resp.).

**Fig. 2:**
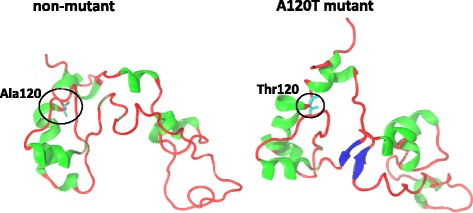
Structural models of N-terminal domain of the non-mutant (right) and A120T mutant (left) ChAT protein

In order to gain a more in-depth view of the effects imparted by rs3810950 polymorphism on the ChAT protein, we have used in silico approach and constructed structural models of N-terminal domain (1st-126th residue) for the increased AD risk-carrying variant A120T and compared it to the structure of other variants. The N-terminal domain of A120T variant appears to be more compact whereas the other variants seem to be less folded suggesting significant changes in the flexibility in the coiled regions (Fig. 2).

## Discussion

Several recent reports, reviews and data analyses have suggested, that rs3810950 polymorphism of the *CHAT* gene could be associated with an increased risk of AD [17, 32, 41, 42]. While some data failed to demonstrate the association [3, 43] other studies supported it [32, 44]. In a Chinese population sample [41] the patients with AD carrying A allele of the rs3810950 polymorphism of *CHAT* gene had a significantly earlier onset of the disease than the patients with G allele (76.4 ± 7.4 vs. 79.2 ± 8.3 years). Another study [42] reported an association between AD and rs3810950 in a group of Korean patients; the results indicated that GA and GA/AA genotypes could be associated with a greater risk of AD in non-ApoE-ε4 allele carriers. The magnitude of the extra risk associated with the genotypes was appreciable (OR equalled 1.639 and 1.630 for GA and GA/AA genotypes, respectively). Oztürk et al. [17] reported a significant association between rs3810950 polymorphism and MMSE scores; comparison of AA homozygous versus AG + GG genotypes produced a strong association, particularly if the data included early onset cases.

Results of a recent meta-analysis [32] have indicated that in three of the five examined genetic models of the rs3810950 polymorphism the subjects with the AA genotype could have a much greater risk of AD than the subjects with the genotypes AG and GG. Another recent meta-analysis [33] revealed significant associations of rs3810950 polymorphism and AD risk both in the overall analysis (GA vs. AA and GG + GA vs. AA) as well as in a specifically ethnically Asian group (GA vs. AA and GG + GA vs. AA).

Thus, while the meta-analyses, overall, favour, the association, neither the design of the individual studies nor their outcomes present a uniform picture which could yield a consistent and large enough set of data conducive to drawing (a) straightforward conclusion(s).

The present results, i.e. the 1.25-fold increase in the risk of AD for the AA genotype, offer a somewhat simpler picture and are in a remarkable accord with those of two other studies. Mubumbila et al. [45] reported that the AA genotype increased the risk of AD with OR = 3.92 and determined odds ratio of 3.7 (3.8 in the present study) for the association between AD and AA genotype. Moreover, this is similar to the results obtained by Lee et al. [46] who reported that the AA genotype increased the risk of AD with OR = 3.92.

In addition, we have noted an increased risk of AD in subjects who had certain combinations of ApoE haplotypes (E3/E3, E3/E4 and E2/E4) and rs3810950 genotypes (cf. Table 3). Most of these relationships are based on modest numbers of patients and controls, though, and may need to be further investigated and verified in much larger studies before any more definite conclusions as to a possible synergy of certain ChAT and ApoE genetic variations in increasing the risk of AD can be drawn.

**Fig. 3:**
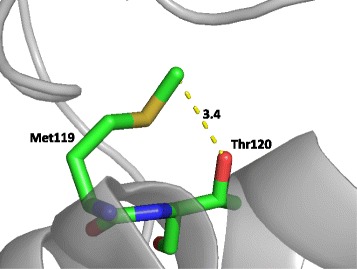
Possible hydrogen bonding between Met119 and Thr120 in A120T mutant with the dotted line corresponding to the distance in ångströms

The central question of the present discussion is – how could a single amino acid change in ChAT molecule associated with the rs3810950 polymorphism result in an increased risk of AD? Our in silico studies provide a possible clue. Dynamics and flexibility of the helical regions in the M isoform of ChAT molecule could be critical for protein-protein interactions [47]. More folded and shorter coil regions together with the presence of possible additional hydrogen bond (Fig. 3) between Thr120 and Met119 in the variant A120T might, therefore, contribute to the putatively lower enzyme activity even if the affected amino acid sequence does not reside within, or, in the vicinity of, the active site of the enzyme [48]. It has been argued that a mutation in *CHAT* gene located at some distance from the sequence of the active site (Val136Met) has a potential to influence the binding of acetyl-CoA and lead to a lower ChAT enzyme activity in addition to having an adverse effect on the enzyme stability thus possibly accounting for the severely reduced expression of ChAT in the affected individual [49]. Such allosteric effects may also exist in the case of rs3810950 but, to our knowledge, they have not yet been investigated. It may be tempting to speculate on how the variation A120T could induce changes in the amount and position of coiled structures in the neighbouring region (N-terminal of the M splice variant) and whether this could affect interactions with cell organelles and/or with other proteins. Additional experiments and more extensive computational studies need to be done to further clarify the role of the A120T change on the ChAT structure before we can adequately understand its impacts on the risk of AD in humans.

Potential limitation of the present study is the presence of multiple ChAT variants in humans and the paucity of the information on their functions (but see [50]), locations and distributions (review: [7]). The current GenBank database lists seven transcript/splice variants of *CHAT* gene. Their expression and distribution in the human brain tissue has not been adequately investigated. This makes it difficult to translate the present findings into more precise structural and functional deficits of specific forms of ChAT which could then be positively linked, taking into account their regional distribution, to AD etiopathogenesis at more intimate, i.e. molecular and cellular level (as has been done e.g. using ChAT molecular modelling in the congenital myastenic syndrome; [51–53]).

## Conclusions

The results of the present study are in line with a growing opinion that ChAT activity is closely linked to AD and can be affected by polymorphisms in *CHAT* gene. The findings suggest that the rs3810950 polymorphism of *CHAT* gene might represent an appreciable risk factor for AD thus providing further support for a causal role of the cholinergic system in the pathogenesis of AD [54]. Significantly, in silico studies pointed to distinct structural characteristics of the ChAT protein which could form a molecular basis for the putative ChAT activity changes associated with the rs3810950 polymorphism and provide directions for future research. Therefore, some 30+ years after the initial formulation of the cholinergic hypothesis of AD, it may be a good time to revisit the hypothesis [5] and hope that it will again contribute to a better understanding of neurodegenerative diseases and help in the development of novel treatments [11].

## Abbreviations

AD: Alzheimer’s disease
Aβ: Amyloid beta
ChAT: Choline acetyltransferase
*CHAT*: Gene for choline acetyltransferase
*SLC18A3*: Gene for VAChT (Solute carrier 18A3)
VAChT: Vesicular acetylcholine transporter

## References

[1] Kidd PM (2008). Alzheimer's disease, amnestic mild cognitive impairment, and age-associated memory impairment: current understanding and progress toward integrative prevention. Altern Med Rev.

[2] Povova J, Ambroz P, Bar M, Pavukova V, Sery O, Tomaskova H (2012). Epidemiological of and risk factors for Alzheimer's disease: a review. Biomed Papers.

[3] Cook LJ, Ho LW, Wang L, Terrenoire E, Brayne C, Evans JG (2005). Candidate gene association studies of genes involved in neuronal cholinergic transmission in Alzheimer's disease suggests choline acetyltransferase as a candidate deserving further study. Amer J Med Gen Part B: Neuropsychiatric Genetics.

[4] Šerý O, Povová J, Míšek I, Pešák L, Janout V (2013). Molecular mechanisms of neuropathological changes in Alzheimer's disease: a review. Folia Neuropathol.

[5] Craig LA, Hong NS, McDonald RJ (2011). Revisiting the cholinergic hypothesis in the development of Alzheimer's disease. Neurosci Biobehav Rev.

[6] Soreq H (2015). Checks and balances on cholinergic signaling in brain and body function. Trends Neurosci.

[7] Oda Y (1999). Choline acetyltransferase: the structure, distribution and pathologic changes in the central nervous system. Pathology Int.

[8] Wevers A (2011). Localisation of pre- and postsynaptic cholinergic markers in the human brain. Behavour Brain Res.

[9] Bellier J-P, Kimura H (2011). Peripheral type of choline acetyltransferase: biological and evolutionary implications for novel mechanisms in cholinergic system. J Chem Neuroanatomy.

[10] Perry E, Walker M, Grace J, Perry R (1999). Acetylcholine in mind: a neurotransmitter correlate of consciousness?. Trends Neurosci.

[11] Contestabile A (2011). The history of the cholinergic hypothesis. Behaviour Brain Res..

[12] Fotiou D, Kaltsatou A, Tsiptsios D, Nakou M (2015). Evaluation of the cholinergic hypothesis in Alzheimer's disease with neuropsychological methods. Aging Clin Exp Res.

[13] Kamkwalala AR, Newhouse PA (2017). Beyond acetylcholinesterase inhibitors: novel cholinergic treatments for Alzheimer's disease. Curr Alzheimer Res.

[14] Davies P, Maloney AJF (1976). Selective loss of central cholinergic neurons in Alzheimer’s disease. Lancet.

[15] Perry RH, Candy JM, Perry EK, Irving D, Blessed G, Fairbairn AD (1982). Extensive loss of choline acetyltransferase activity is not reflected by neuronal loss in the nucleus of Meynert in Alzheimer’s disease. Neurosci Lett.

[16] Nagai T, McGeer PL, Peng EG, McGeer EG, Dolman CE (1983). Choline acetyltransferase immunohistochemistry in brains of Alzheimer’s disease patients and controls. Neurosci Lett.

[17] Oztürk A, DeKosky ST, Kamboh MI (2006). Genetic variation in the choline acetyltransferase (CHAT) gene may be associated with the risk of Alzheimer's disease. Neurobiol Aging.

[18] Bierer LM, Haroutunian V, Gabriel S, Knott PJ, Carlin LS, Purohit Dushyant P, Perl DP, Schmeidler J, Kanof P, Davis KL (1995). Neurochemical correlates of dementia severity in Alzheimer’s disease: relative importance of the cholinergic deficits. J Neurochem.

[19] Winick-Ng W, Caetano FA, Winick-Ng J, Morey TM, Heit B, Rylett RJ (2016). 82-kDa choline acetyltransferase and SATB1 localize to β-amyloid induced matrix attachment regions. Sci Rep.

[20] Nunes-Tavares N, Santos LE, Stutz B, Brito-Moreira J, Klein WL, Ferreira ST (2012). Inhibition of choline acetyltransferase as a mechanism for cholinergic dysfunction induced by amyloid-β peptide oligomers. J Biol Chem.

[21] Hawley WR, Witty CF, Daniel JM, Dohanich GP (2015). Choline acetyltransferase in the hippocampus is associated with learning strategy preference in adult male rats. Behaviour Brain Res.

[22] Park D, Yang YH, Bae DK, Lee SH, Yang G, Kyung J (2013). Improvement of cognitive function and physical activity of aging mice by human neural stem cells over-expressing choline acetyltransferase. Neurobiol Aging.

[23] Iachini I, Iavarone A, Senese VP, Ruotolo F, Ruggiero G (2009). Visuospatial memory in healthy elderly, AD and MCI: a review. Curr Aging Sci.

[24] Shin K, Cha Y, Kim KS, Choi EK, Choi Y, Guo H, et al. Human neural stem cells overexpressing choline acetyltransferase restore unconditioned fear in rats with amygdala injury. Behav Neurol. 2016; 10.1155/2016/8521297. 10.1155/2016/8521297 PMC4819097 27087745

[25] Fgaier H, Mustafa IH, Awad AA, Elkamel A. Modeling the interaction between β-amyloid aggregates and choline acetyltransferase activity and its relation with cholinergic dysfunction through two-enzyme/two-compartment model. Comp Math Methods Med. 2015; 10.1155/2015/923762. 10.1155/2015/923762 PMC4568057 26413144

[26] Allaway KC, Machold R (2017). Developmental specification of forebrain cholinergic neurons. Dev Biol.

[27] Matsuo A, Bellier JP, Nishimura M, Yasuhara O, Saito N, Kimura H (2011). Nuclear choline acetyltransferase activates transcription of a high-affinity choline transporter. J Biol Chem.

[28] Resendes MC, Dobransky T, Ferguson SS, Rylett RJ (1999). Nuclear localization of the 82-kDa form of human choline acetyltransferase. J Biol Chem.

[29] Ohno K, Tsujino A, Brengman JM, Harper CM, Bajzer Z, Udd B (2001). Choline acetyltransferase mutations cause myasthenic syndrome associated with episodic apnea in humans. Proc Nat Acad Sci.

[30] Gill SK, Ishak M, Dobransky T, Haroutunian V, Davis KL, Rylett RJ (2007). 82-kDa choline acetyltransferase is in nuclei of cholinergic neurons in human CNS and altered in aging and Alzheimer disease. Neurobiol Aging.

[31] Misawa H, Matsura J, Oda Y, Takahashi R, Deguchi T (1997). Human choline acetyltransferase mRNA with different 5′-region produce a 69-kDa major translation product. Mol Brain Res.

[32] Liu Y, Chen Q, Liu X, Dou M, Li S, Zhou J, et al. Genetic association of CHAT rs3810950 and rs2177369 polymorphisms with the risk of Alzheimer's disease: a meta-analysis. BioMed Res Internat. 2016:9418163. https://www.ncbi.nlm.nih.gov/pubmed/27597977. 10.1155/2016/9418163 PMC5002460 27597977

[33] Yuan H, Xia Q, Ling K, Wang X, Wang X, Du X (2016). Association of Choline Acetyltransferase Gene Polymorphisms (SNPs rs868750G/a, rs1880676G/a, rs2177369G/a and rs3810950G/a) with Alzheimer's disease risk: a meta-analysis. PLoS One.

[34] Janošíková B, Zavadáková P, Kožich V (2005). Single-nucleotide polymorphisms in genes relating to homocysteine metabolism: how applicable are public SNP databases to a typical European population?. Eur J Human Gen.

[35] Šerý O, Hlinecká L, Balcar VJ, Janout V, Povová J (2014). Diabetes, hypertension and stroke–does Alzheimer protect you?. Neuroendocrinol Lett.

[36] Šerý O, Hlinecká L, Povová J, Bonczek O, Zeman T, Janout V (2016). Arachidonate 5-lipoxygenase (ALOX5) gene polymorphism is associated with Alzheimer's disease and body mass index. J Neurol Sci.

[37] Šerý O, Janoutová J, Ewerlingová L, Hálová A, Lochman J, Janout V (2017). CD36 gene polymorphism is associated with Alzheimer's disease. Biochimie.

[38] R Foundation for Statistical Computing, A language and environment for statistical computing, http://www.R-project.org/ 2015 (accessed 12.Nov.2015).

[39] Šali A, Blundell TL (1993). Comparative protein modelling by satisfaction of spatial restraints. J Mol Biol.

[40] Humphrey W, Dalke A, Schulten K (1996). VMD: Visual molecular dynamics. J Mol Graph.

[41] Tang M, Rao D, Ma C, Guo Y, Han H, Ling K (2008). Evaluation of choline acetyltransferase gene polymorphism (2384 G/a) in Alzheimer’s disease and mild cognitive impairment. Dement Geriat Cognit Disorders.

[42] Jo SA, Ahn K, Kim JH, Kang BH, Kim E, Jo I (2006). ApoE-ε 4-dependent association of the choline acetyltransferase gene polymorphisms (2384G> a and 1882G> a) with Alzheimer's disease. Clin Chimica Acta.

[43] Schwarz S, Eisele T, Diehl J, Müller U, Förstl H, Kurz A (2003). Lack of association between a single nucleotide polymorphism within the choline acetyltransferase gene and patients with Alzheimer's disease. Neurosci Lett.

[44] Gao L, Zhang Y, Deng J, Yu W, Yu Y (2016). Polymorphisms of CHAT but not TFAM or VR22 are associated with Alzheimer disease risk. Med Sci Monit.

[45] Mubumbila V, Sutter A, Ptok U, Heun R, Quirin-Stricker C (2002). Identification of a single nucleotide polymorphism in the choline acetyltransferase gene associated with Alzheimer's disease. Neurosci Lett.

[46] Lee JJ, Jo SA, Park JH, Lee SB, Jo I, Huh Y (2012). Choline acetyltransferase 2384G> a polymorphism and the risk of Alzheimer disease. Alz Dis Assoc Dis.

[47] Azzarito V, Long K, Murphy NS, Wilson MJ (2013). Inhibition of α-helix-mediated protein-protein interactions using designed molecules. Nat Chem.

[48] Cai Y, Cronin CN, Engel AG, Ohno K, Hersh LB, Rodgers DW (2004). Choline acetyltransferase structure reveals distribution of mutations that cause motor disorders. EMBO J.

[49] Arredondo J, Lara M, Gospe SM, Mazia CG, Vaccarezza M, Garcia-Erro M (2015). Choline acetyltransferse mutations causing congenital myasthenic syndrome: molecular findings and genotype-phenotype correlations. Human Mutat.

[50] Liu X, Shi Y, Niu B, Shi Z, Li J, Ma Z (2016). Polymorphic variation in CHAT gene modulates general cognitive ability: an association study with random student cohort. Neurosci Lett.

[51] Morey TM, Albers S, Shilton BH, Rylatt RJ (2016). Enhanced ubiquitination and proteasomal degradatin of catalytically deficient human choline acetyl transferase mutants. J Neurochem.

[52] Dobransky T, Rylett RJ (2005). A model for dynamic regulation of choline acetyltransferase by phophorylation. J Neurochem.

[53] Shen XM, TO C, Brengman J, Acsadi G, Iannaconne S, Karaca E (2011). Functional consequences and structural interpretation of mutations of human choline acetyltransferase. Human Mutat..

[54] Schliebs R, Arendt T (2011). The cholinergic system in aging and neuronal degeneration. Behaviour Brain Res..

